# The Effectiveness of Cucurbitacin B in BRCA1 Defective Breast Cancer Cells

**DOI:** 10.1371/journal.pone.0055732

**Published:** 2013-02-05

**Authors:** Moltira Promkan, Sumana Dakeng, Subhas Chakrabarty, Oliver Bögler, Pimpicha Patmasiriwat

**Affiliations:** 1 Center for Innovation Development and Technology Transfer, Faculty of Medical Technology, Mahidol University, Salaya, Nakhon Pathom, Thailand; 2 Department of Medical Microbiology, Immunology and Cell Biology. Southern Illinois University School of Medicine and Simmons Cancer Institute, Springfield, Illinois, United States of America; 3 Department of Neurosurgery, The University of Texas M. D. Anderson Cancer Center, Houston, Texas, United States of America; 4 Department of Clinical Microscopy, Faculty of Medical Technology, Mahidol University, Salaya, Nakhon Pathom, Thailand; University of California Irvine, United States of America

## Abstract

Cucurbitacin B (CuB) is one of the potential agents for long term anticancer chemoprevention. Cumulative evidences has shown that cucurbitacin B provides potent cellular biological activities such as hepatoprotective, anti-inflammatory and antimicrobial effects, but the precise mechanism of this agent is not clearly understood. We examine the biological effects on cancer cells of cucurbitacin B extracted from a Thai herb, *Trichosanthes cucumerina* L. The wild type (wt) BRCA1, mutant BRCA1, BRCA1 knocked-down and BRCA1 overexpressed breast cancer cells were treated with the cucurbitacin B and determined for the inhibitory effects on the cell proliferation, migration, invasion, anchorage-independent growth. The gene expressions in the treated cells were analyzed for p21/^Waf1^, p27^Kip1^ and survivin. Our previous study revealed that loss of BRCA1 expression leads to an increase in survivin expression, which is responsible for a reduction in sensitivity to paclitaxel. In this work, we showed that cucurbitacin B obviously inhibited knocked-down and mutant BRCA1 breast cancer cells rather than the wild type BRCA1 breast cancer cells in regards to the cellular proliferation, migration, invasion and anchorage-independent growth. Furthermore, forcing the cells to overexpress wild type BRCA1 significantly reduced effectiveness of cucurbitacin B on growth inhibition of the endogenous mutant BRCA1 cells. Interestingly, cucurbitacin B promotes the expression of p21/^Waf1^ and p27^Kip1^ but inhibit the expression of survivin. We suggest that survivin could be an important target of cucurbitacin B in BRCA1 defective breast cancer cells.

## Introduction

Cucurbitacins are tetracyclic triterpenes isolated from plant in the Cucurbitaceae families that has been used in traditional medicine for centuries [1], [2]. Cucurbitacins have potential to be used as a favorable phytochemical for cancer prevention [3] and the compounds continue to be structural improvement for the future chemotherapeutic approach. However, the mechanism of antitumor activity of cucurbitacins in breast cancer remains unclear. Previous studies showed that some of these compounds have a broad range of biological effects, including anti-inflammatory, hepatoprotective and anticancer activities [4]–[10]. Cucurbitacins are highly diverse and arbitrarily divided into twelve types, the cucurbitacin A to T [1]. Several types of cucurbitacin compounds have been studied *in vitro* and *in vivo* for their anticancer effects. For example, cucurbitacin E treatment can inhibit the viability of pancreatic cancer cells (PANC-1) and induce apoptosis via suppression of STAT3 phosphorylation and up-regulation of p53 [8]. Cucurbitacin E also inhibits the proliferation of prostate cancer cells and causes disruption of the cytoskeleton structure of actin and vimentin [11]. Cucurbitacin I was shown to inhibit nasopharyngeal carcinoma cell (NPC) proliferation and invasion, and also inhibit NPC tumor formation in nude mice [7]. Similar to cucurbitacin E, cucurbitacin I can also inhibit STAT3 phosphorylation [12]. Cucurbitacin B is found in many Cucurbitaceae species and it is one of the abundant forms of cucurbitacins [1], [13]. In breast cancer cell lines, cucurbitacin B and E glucoside combination as well as each of them can induce cell-cycle arrest in the G_2_/M phase by reducing the amount of p34^CDC2^/cyclin B1 complex [14]. Cucurbitacin glucoside treatment caused changes in the overall breast cancer cell morphology from elongated to a round-shaped cell, indicating the impairment of actin filament organization [14]. As found in the other cucurbitacins, cucurbitacin B has been reported as the antiproliferative agent of breast cancer cells *in vitro* and *in vivo* [15] and can induce apoptosis in Bcap37 breast cancer cells [16]. Our previous work revealed that cucurbitacin B inhibits growth and telomerase activity in breast cancer cell lines (T47D, SKBR-3, and MCF-7) and the inhibitory effect was obviously seen in the estrogen receptor (ER)-negative breast cancer cells SKBR-3 [17]. It also inhibits hTERT and c-Myc protein expression. These findings imply that cucurbitacin B exerts an anticancer effect by inhibiting telomerase via down regulating both the hTERT and c-Myc expression in breast cancer cells.

Hereditary breast cancer accounts for 5–10% of all breast cancers [18], [19]. *BRCA1* and *BRCA2* are tumor suppressor genes in which loss or inactivation increases the risk of hereditary breast and ovarian cancer [19], [20]. BRCA1 is a multifunctional protein which interacts with various proteins in the nucleus to play roles in DNA repair, transcriptional regulation and maintenance genome stability [20], [21]. Thus, loss of BRCA1 function may lead to accumulation of chromosomal damage, abnormality in growth control and finally tumorigenesis [22]. Sixty-five percents of Thai familial and early-onset breast/ovarian cancer exhibited *BRCA1/2* mutations within coding region [23]. The exonic mutation was 44% cancer related frameshift mutation while 21% was missense mutation. [23], [24].

Two *BRCA1* mutations found in high risk breast/ovarian cancer families in Thailand are missense mutation in exon 11 in which the bases change from T to C at nucleotide 2685 and nonsense mutation of deleted A at nucleotide 3300. The two mutations cause amino acid changes from Tyrosine to Histidine in codon 856 and the stop site at codon 1061, respectively [23]. These two mutations might interfere with the gene functions and could be resulted in an increased risk of cancer.

The presence or absence of functional BRCA1 has a significant effect on the cellular proliferation as well as the response to chemotherapy. BRCA1 is therefore suggested to be a potential predictive biomarker in the treatment of breast cancer [25]. BRCA1 has shown to regulate sensitivity of cancer cells to some chemotherapeutic agents. The lack of BRCA1 with deficient DNA repair results in increased sensitivity to DNA damage-based chemotherapeutics, while the presence of BRCA1 promotes sensitivity to antimicrotubule agents probably through modulation of cell cycle and apoptosis [25]. We recently reported that BRCA1 down-modulates the malignant behavior of breast cancer cells in regard to cell proliferation, migration, invasion and anchorage-independent growth. BRCA1 promotes the expression of the cell cycle check point proteins p21/^Waf1^ and p27^Kip1^ and inhibits the expression of an anti-apoptotic protein survivin [26]. Loss of BRCA1 expression leads to an increase in survivin expression, leading to reduce paclitaxel sensitivity [26]. This drug is highly cytotoxic to breast cancer cells which are dued to its interference with microtubule function as well as apoptotic induction [27]–[29]. Apart from the role of survivin in malignant progression, this factor also plays a crucial role in blocking drug-induced apoptosis and hence it is a critical determinant of drug resistance [30], [31].

Cell migration, invasion and growth in an anchorage independent are the characteristics of malignant tumors [32]. Survival rate of patients can exceed up to 90 percent when breast carcinoma still remains in breast tissue. However, long term survival and curable rate decrease as the cancer cells have metastasized. Thus, high effective treatment is necessary in order to increase survival rate and prevent tumors metastasis. In this work, we report the biological effects of cucurbitacin B extracted from medicinal herb *Trichosanthes cucumerina* L. [33], on human breast cancer cells with or without functional BRCA1. The malignant properties regarding to cellular proliferation, migration, invasion, anchorage-independent growth, expression of p21/^Waf1^, p27^Kip1^ and survivin in the breast cancer cells are reported herein.

## Materials and Methods

### Plant material and cucurbitacin purification

Isolation of cucurbitacins from fruit of *Trichosanthes cucumerina* L. was performed as described previously [33]. All necessary permits were obtained for the described field studies. In this study the purified cucurbitacin B was dissolved in 1% dimethylsulfoxide (DMSO) and diluted with Dulbecco's modified Eagle's medium (DMEM) (Sigma, St. Louis, MO) to the desired concentrations prior to use.

### Cell culture

BRCA1 wild type cells (MCF-7, MDA-MB-231) and BRCA1 mutant cells (HCC1937, MDA-MB-436 [34]) were purchased from the American Type Culture Collection (ATCC). All cell lines were cultured in DMEM (Sigma, St. Louis, MO) supplemented with sodium bicarbonate, peptone, vitamins, amino acids and 5% fetal bovine serum. All cells were cultured at 37°C in 5% CO_2_ humidified atmosphere.

### Vector constructions, transfection, selection and development of stable transfectants

Plasmid of shRNA-BRCA1 expression vector targeting BRCA1 and its corresponding scrambled control vector were constructed as previously described [26]. Plasmids of shRNA-BRCA1 or shRNA-scrambled control were transfected into the cells with endogenous wild type BRCA1 in order to knock down the gene expression. Stable BRCA1 knocked-down or shRNA-scrambled control transfectants were established as previously described [26]. Transfectants were cultured in DMEM medium containing 1 µg/ml of puromycin.

A plasmid vector of BRCA1 splice variant, in which absence of exon 9 and 10 (designated as BRCA1 Delta(9,10)), was created by cloning the variant BRCA1 cDNA into the pcDNA3.1 expression vector using artificially engineered 5′ HindIII and 3′ XhoI sites. The BRCA1 cDNAs were contributed by Mien-Chie Hung (The University of Texas M. D. Anderson Cancer Center, Houston, TX). cDNA encoding the BRCA1 Delta(9,10) protein was subcloned into pCEP4 under the CMV promoter (pCEP4-BRCA1-Delta(9,10)). This vector contains Tag2 which allows expression of the protein with an amino-terminal FLAG sequence.

In order to obtain vector for wild type BRCA1 with full length expression, we amplified cDNA of exon 9–10 of BRCA1 from the mammary epithelium cells HBL-100, using forward primer (5′-GAA CAG AAA GAA ATG GAT TTA TCT-3′) and reverse primer (5′-GAC CCA GAG TGG GCA GA-3′). The specified cDNA region was then subcloned into pCEP4-BRCA1 Delta(9,10) at the 5′ SphI and 3′ BbvCI sites.

### PCR-Based site directed mutagenesis

#### BRCA1-3300delA

The nonsense mutation BRCA1-3300delA vectors were created by modification of pCEP4-BRCA1 full length plasmid. In brief, amplification with twelve PCR cycles (95°C for 5 min, 12 cycles of 95°C for 45 sec, 68°C for 1 min, and 72°C for 1 min and final extension at 72°C for 10 min) was performed using platinum Taq DNA polymerase and 10 µM of oligonucleotide primers (3300delA; forward primer 5′-ATT AAT GAA TAG GTT CCA GTG ATG AAA AC-3′, reverse primer 5′-CTG GAA CCT ATT CAT TAA TAC TGG AG-3′). One µl of *Dpn I* restriction enzyme (10 U/µl) was added directly to each PCR reaction. Each reaction mixture was gently mixed by up and down pipetting then spinned down in for 1 minute and each reaction was immediately incubated at 37°C for 1 hour to digest the parental supercoiled dsDNA. The PCR product was purified and transformed in E.coli. After then, the cells were plated on ampicillin LB agar plate containing X-gal and incubated for 16 hours at 37°C. White clones were recovered and re-streaked on master plates. Cells containing the plasmid with mutated BRCA1 inserts generated the white clones, from which the DNA was then extracted and sequenced. BRCA1 3300delA plasmids were stably transfected into the BRCA1-defective breast cancer cell line MDA-MB-436.

#### Missense mutation (Tyr856His)

The expression vector containing missense mutation (Tyr856His) BRCA1 was created by modification of the pCEP4-BRCA1 plasmid as a template and the mutagenesis was carried out in the same procedure as above, using the following PCR primers; Tyr856His; forward primer 5′-TCA GCA TTT GCA GAA TAC ATT CAA GGT -3′, reverse primer 5′- GCT TTG AAA CCT TGA ATG TAT TCT GC -3′. The resulting mutant plasmids containing BRCA1 (Tyr856His) were stably transfected into MCF-7 and MDA-MB-231 cells harboring endogenous wild type BRCA1.

The transfected cells were studied for their cellular capability of proliferation, migration, invasion and anchorage-independent growth. For confirming *BRCA1* sequence, the GenBank accession number U14680 was used as the reference database.

### MTS assay

The CellTiter 96® AQueous Non-Radioactive Cell Proliferation Assay is a colorimetric method for determining the number of viable cells in proliferation as well as chemosensitivity assays. This assay is composed of solutions containing a tetrazolium compound [3-(4,5-dimethylthiazol-2-yl)-5-(3-carboxymethoxyphenyl)-2-(4-sulfophenyl)-2H-tetrazolium, inner salt, MTS] and an electron coupling reagent (phenazine methosulfate, PMS). MTS is bioreduced by cells into a formazan product that is soluble in tissue culture medium. This MTS assay was performed using a CellTiter96TMA Queous Assay (MTS) kit, according to the manufacturer's instructions (Promega, Madison, WI). Briefly, 1×10^5^ cells were seeded into each well of 96-well culture plates and incubated at 37°C in CO_2_ incubator for 24 h. The medium was then replenished with medium in the absence or presence of cucurbitacin B. Cells were then incubated at 37°C in CO_2_ incubator for 24 h, 48 h, 72 h and 96 h. The cells were then rinsed with plain DMEM medium followed by adding CellTiter96™ Aqueous One Solution Reagent to the culture wells. The absorbance at 490 nm was then recorded using a Multiskan Specturm (Thermo Electron Corporation, Waltham, MA). The tested results represent the mean and standard error of three experiments.

### Cytotoxicity assay with trypan blue

The trypan blue dye exclusion test was determined for the cytotoxic response to paclitaxel [26]. Cells were seeded at a density of 1×10^5^ cells/well in 24-well plates and incubated overnight in a 5% CO_2_ humidified incubator. The cells were then either treated with 4 µM paclitaxel (Sigma, St. Louis, MO) or with various concentrations of cucurbitacin B. After 48 h of continuous drug exposure, cells were detached by trypsinization and the number of viable cells were determined using a Beckman Coulter Vi-cellTM XR cell viability automated cell counting analyzer (Beckman Coulter, Inc., Miami, FL). Cytotoxic responses to the drugs were expressed as the percent of cells killed as compared with the control cells. Triplicate determinations were performed in each experiment and the results represent the mean and standard error of three experiments.

### Anchorage-independent growth assay

Soft agar colony formation assays were performed in 60 mm culture dishes as described previously [26]. Briefly, cells (5×10^3^) were suspended in 1.5 ml of 0.3% agar (Sigma, St. Louis, MO) in complete growth medium containing DMEM supplemented with 10% FBS in the absence or presence of cucurbitacin B. The cell suspension was then added to a base layer of 0.6% agar containing medium and incubated at 37°C in a 5% CO_2_ humidified incubator for 2 weeks. Colonies were stained by adding 500 µl of 2 mg/ml P-iodonitrotetrazolium violet (Sigma, St. Louis, MO) to the culture dishes. The dishes were then incubated at 37°C for 24 hours. Stained colonies were manually counted under a magnifying lens. The number of colonies presented from each phenotype represents the mean and standard error of triplicate determinations.

### Cell migration and invasion assay

These assays were performed as described previously [26]. *In vitro* cell migration assay was performed using Transwell chambers with 8 µl pore size (Costar Corning, Corning, NY). Actively growing cells of approximately 70% confluence in culture flasks were allowed to adapt in serum-free medium for 24 h. These cells were then detached by trypsinization, washed with PBS and re-suspended in serum-free culture medium in the absence or presence of cucurbitacin B. Total volume of 100 µl cell suspension (1×10^6^ cells/ml) was added to each upper chamber and the serum containing culture medium was added to the bottom chambers. The cells were then incubated for 24 h at 37°C. Cells that had migrated into the bottom chambers were fixed with 4% formaldehyde in PBS and the number of cells was counted under a phase contrast microscope. Triplicate determinations were performed in each experiment. Invasion assays were performed in a similar manner, using Matrigel-coated membrane inserted between the two chambers.

### Western blotting

Cells were seeded into 6-well plates and treated with 15 µg/ml cucurbitacin B for 48 h. The cells were prepared for western blotting as previously described [26], [35]. Briefly, cell lysates were prepared in the solution containing lysis buffer [50 mM Tris (pH 7.5), 200 mM KCl, 1 mM EDTA, 1 mM EGTA, 1% NP40, 1 mM PMSF, 5 mM sodium orthovanadate, 10 µl/ml protease inhibitor cocktail (EMD chemicals, San Diego, CA)]. Protein concentrations were determined by the Bio-Rad dye-binding assay (Bio-Rad, Hercules, CA). Cellular proteins were fractionated by SDS-PAGE and transferred onto polyvinylidene difluoride (PVDF) membrane. The membrane was blocked (with 5% non-fat dried milk in 1× TBS buffer containing 0.1% Tween 20) and immunostained with primary antibodies. Anti-BRCA1 (Cell Signaling Technology, Danvers, MA; 1∶1000), anti-p21^/Waf1/cip1^ (Cell Signaling; 1∶2000), anti-p27 (Santa Cruz biotechnology, Santa Cruz, CA; 1∶2000) or anti-human survivin antibodies (R&D Systems, Minneapolis, MN; 1∶3000) were used as primary antibodies. Horseradish peroxidase-conjugated secondary antibodies in conjunction with chemiluminescence detection in the FUJIFILM LAS-3000 system (Fujifilm Life Science, Stanford, CT) were used to visualize the binding of specific primary antibodies to immobilized proteins. Quantitative analysis of protein expression was performed using a Multi-Gauge Image software installed in the FUJIFILM LAS-3000 system. Changes in protein expression were calculated by comparison to the control. The numbers on the blots represent relative values of protein expression levels as compared to the control. GAPDH expression [using anti-human GAPDH antibody (Abcam, Cambridge, MA)] was used as internal control.

### Statistical analysis

Statistically difference between the two groups was assessed using student's t-test. P value of less than 0.01 was considered as significant different. The percentage of cells inhibition after cucurbitacin B treatment was calculated by the following formula:
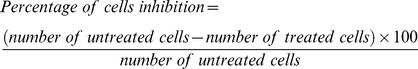



## Results

### Cucurbitacin B inhibits cellular proliferation, migration, invasion and anchorage-independent growth

**Figure 1:**
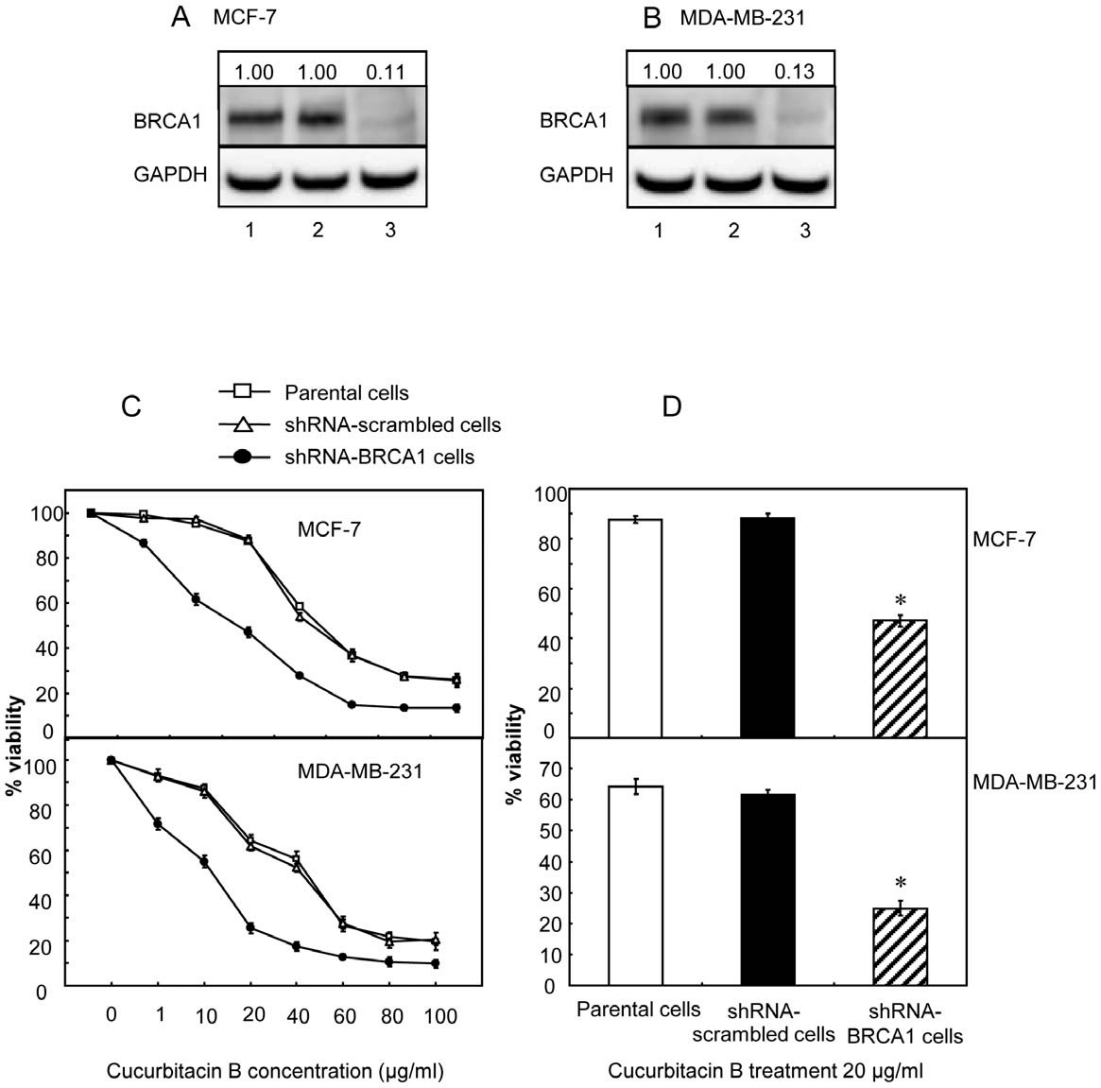
Cell viability of cucurbitacin B treated BRCA1 knocked-down breast cancer cells. (A) and (B), BRCA1 expressions were knocked down in MCF-7 cells and MDA-MB-231 cells, respectively. Lanes 1 and 2, parental and transfected control cells. Lanes 3, shRNA-BRCA1 transfected cells. GAPDH expression represents internal control. All the densitometric values were normalized to loading controls (GAPDH), and the fold change relative to the parental control are indicated numerically above the blots. (C), Cells were treated with 1, 10, 20, 40, 60, 80 and 100 µg/ml cucurbitacin B for 48 h. Three repetitive experiments were done and each was run in triplicate. (D), The results from the experiment shown in (C) are compared in each group at the specified concentration of 20 µg/ml cucurbitacin B. Knocked-down BRCA1 breast cancer cells showed significant higher sensitivity to cucurbitacin B when compared to the parental cells, (* *p*<0.01).

We determined the roles of BRCA1 as a factor that might influence the action of cucurbitacin B on cell proliferation, anchorage-independent growth, cell migration and invasion. Loss or reduced BRCA1 expression could lead to a significant increase in cellular proliferation. The wt-BRCA1 breast cancer cells (MCF-7 and MDA-MB-231) were transfected with shRNAs targeting to BRCA1. The BRCA1 protein was significantly reduced in cells stably transfected with the shRNA-BRCA1 plasmid but not in the cells transfected with the control vector (Fig. 1A, 1B). The IC_50_ of cucurbitacin B in the parental MCF-7 and MDA-MB-231 cells, their shRNA-BRCA1 transfected cells and vector control cells were analyzed. Each group of cells were incubated with specified concentrations of cucurbitacin B, ranging from 1 to 100 µg/ml and the cells were assessed for their viability using MTS assay at 48 h post incubation. The growth inhibitory effect of cucurbitacin B was shown in Figure 1C. Table 1 details the IC_50_ of cucurbitacin B in each group. It is obvious that the growth of knocked-down BRCA1 cells were inhibited by cucurbitacin B more than the parental and scrambled control cells. A significant decrease in cell viability of knocked-down BRCA1 cells after treating with 20 µg/ml cucurbitacin B is shown in Figure 1D.

**Table 1:** The half maximal inhibitory concentration (IC_50_) in each group of breast cancer cells.

Cell lines		IC50 (µg/ml)
MCF-7		
	wt-BRCA1 parental cells	48.6
	shRNA-scrambled control cells	47.6
	shRNA-BRCA1 cells (knocked-down)	19.3
MDA-MB-231		
	wt-BRCA1 parental cells	38.9
	shRNA-scrambled control cells	36.7
	shRNA-BRCA1 cells (knocked-down)	12.9
HCC1937		
	mutated BRCA1 cells (5382insC)	18.4
MDA-MB-436		
	mutated BRCA1 cells (5396+1G>A)	12.1

**Figure 2:**
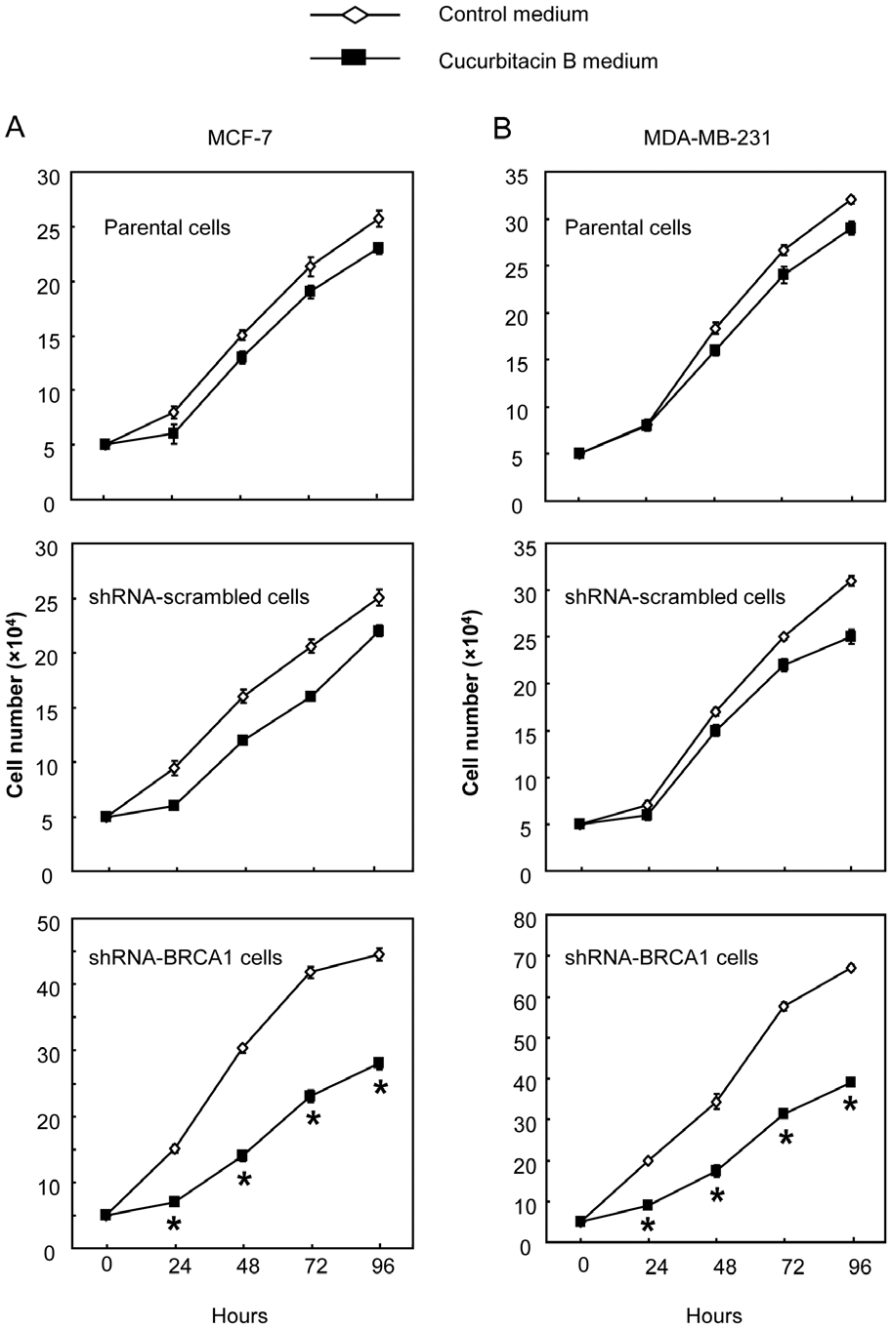
Cell proliferations of MCF-7 (A) and MDA-MB-231 (B) after treatment with 12 µg/ml cucurbitacin B into the culture medium in the parental cells, transfected control (shRNA-scrambled) cells and shRNA-BRCA1 knocked-down cells. Each experiment was performed in triplicate. ShRNA-BRCA1 knocked-down cells showed significant highly sensitive to cucurbitacin B when compared to the BRCA1 parental cells, (* *p*<0.01).

We further examined the proliferative rate in each group of cells after treated with 12 µg/ml of cucurbitacin B. The differences in the rate of proliferation were obviously seen as early as 24 hours, and the differences were further increased over a four-day culture period (Fig. 2A, 2B). Cucurbitacin B untreated controls of MCF-7 and MDA-MB231 cells with BRCA1 functional loss (shRNA-BRCA1, Figure 2-bottom) proliferated approximately twice faster than the parental and shRNA scrambled counterparts having wild type BRCA1 (upper and middle). Addition of cucurbitacin B into the culture medium effectively restrained growth of shRNA-BRCA1 (knocked-down) cells. In Figure 2, the cell numbers counted from 24 h to 72 h indicated that cucurbitacin B treatment to these BRCA1 knocked down cancer cells could impede growth for approximately 50 percent as compared to the fast growing, untreated BRCA1 knocked-down partner. Proliferation of cucurbitacin B treated parental cells and scrambled cells harboring the functional (wt) BRCA1 tumor suppressor was mildly reduced when compared to its paired untreated control cells. Molecules thought to be vulnerable by the effect of cucurbitacin B were addressed and details in the following results.

**Figure 3:**
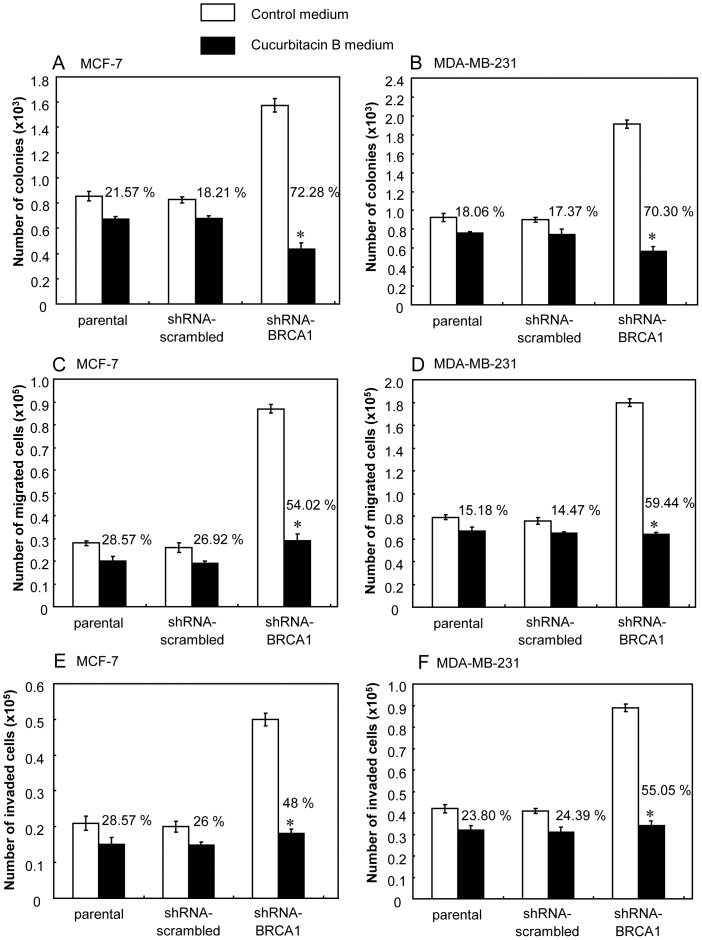
The clonal anchorage-independent growth, cell migration and invasion after treatment with 12 µg/ml cucurbitacin B. (A) and (B), Anchorage-independent growth with or without cucurbitacin B treatment in each group of the MCF-7 and MDA-MB-231 cells. (C) and (D), The capability of cell migration in the MCF-7 and MDA-MB-231. (E) and (F), The invasive capability of the MCF-7 and MDA-MB-231, respectively. (* *p*<0.01).

Loss or reduced BRCA1 expression led to an increase in malignant behavior in terms of the propensity for anchorage-independent growth, cell migration and invasion of matrigel [26]. Clonogenic assay relies on individual colony forming cell to proliferate in soft agar culture to form a colony. When breast cancer cells were cultured in soft agar either with or without cucurbitacin B, clonal growth of the BRCA1 knocked-down cells was inhibited significantly in the presence of cucurbitacin B compared with the untreated control cells. The clonal growth, as determined by the number of colonies formed in soft agar, was reduced by cucurbitacin B (Fig. 3A, 3B) and decrease in the size of colonies was also observed in the cucurbitacin B treated culture (not shown). Cucurbitacin B significantly inhibited cellular migration and invasion in the shRNA-BRCA1 transfected cells but had no effect upon parental cells at concentration of 12 µg/ml (Fig. 3C–3F). These results indicate that the biological action of cucurbitacin B in cancer cells could be associated with the BRCA1 function.

### Cucurbitacin B induced expression of p27^Kip1^ and p21/^Waf1^ but suppressed the expression of survivin in BRCA1 dependent manner

**Figure 4:**
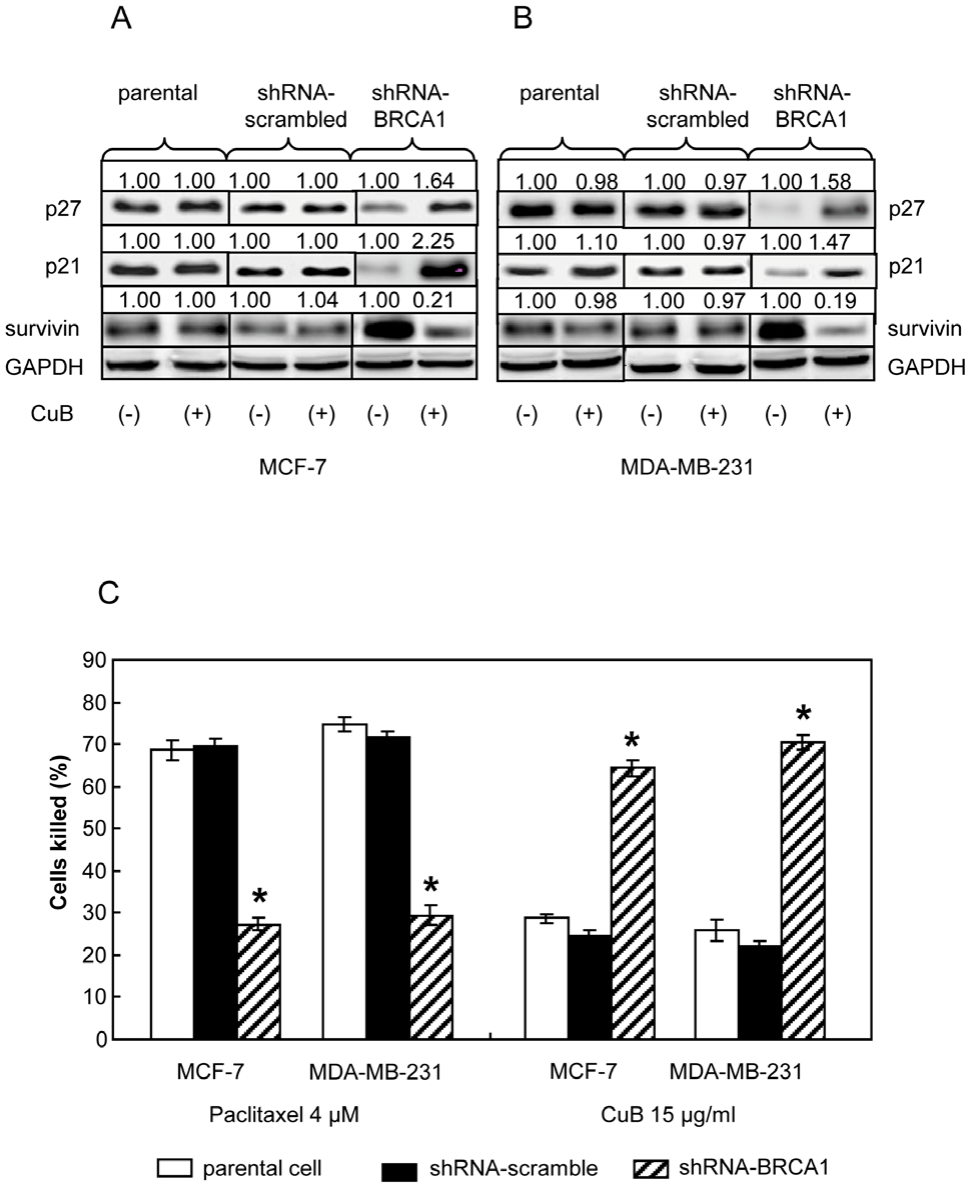
Cucurbitacin B treatments in the parental MCF-7 and MDA-MB-231 (endogenously expressed wt-BRCA1), BRCA1 knocked-down counterparts and shRNA scrambled controls. (A) and (B), Western blot analysis for p21, p27 and survivin proteins after the cells were cultured either in control medium or in the medium containing 15 µg/ml cucurbitacin B for 48 h. GAPDH was used as loading control. (C), The degrees of significantly cytotoxic responses (indicating as percent killed) to paclitaxel and cucurbitacin B in wt-BRCA1 parental and BRCA1 knocked-down MCF-7 and MDA-MB-231 cells are shown, (* *p*<0.01).

Knocking down BRCA1 in breast cancer cells resulted in an increase in the expression of survivin which associated with malignant progression and drug resistance [26]. In the absence of cucurbitacin B treatment, knocking down of BRCA1 expression could result in an increased anti-apoptotic molecule survivin expression with a concurrent reducdion of paclitaxel sensitivity (Fig. 4A, 4B and 4C). Treatment of the BRCA1 knocked-down cells with 15 µg/ml cucurbitacin B could induce cell cycle inhibitors p27^Kip1^ and p21/^Waf1^ expression but down modulate survivin expression (Fig. 4A, 4B). Reduced expression of survivin in these cucurbitacin B treated cells could be an important sign of increased apoptotic process, as a significant increased sensitivity to cucurbitacin B was observed (Fig. 4C).

### BRCA1 mutant cells are more sensitive to cucurbitacin B than the non-mutant counterpart

**Figure 5:**
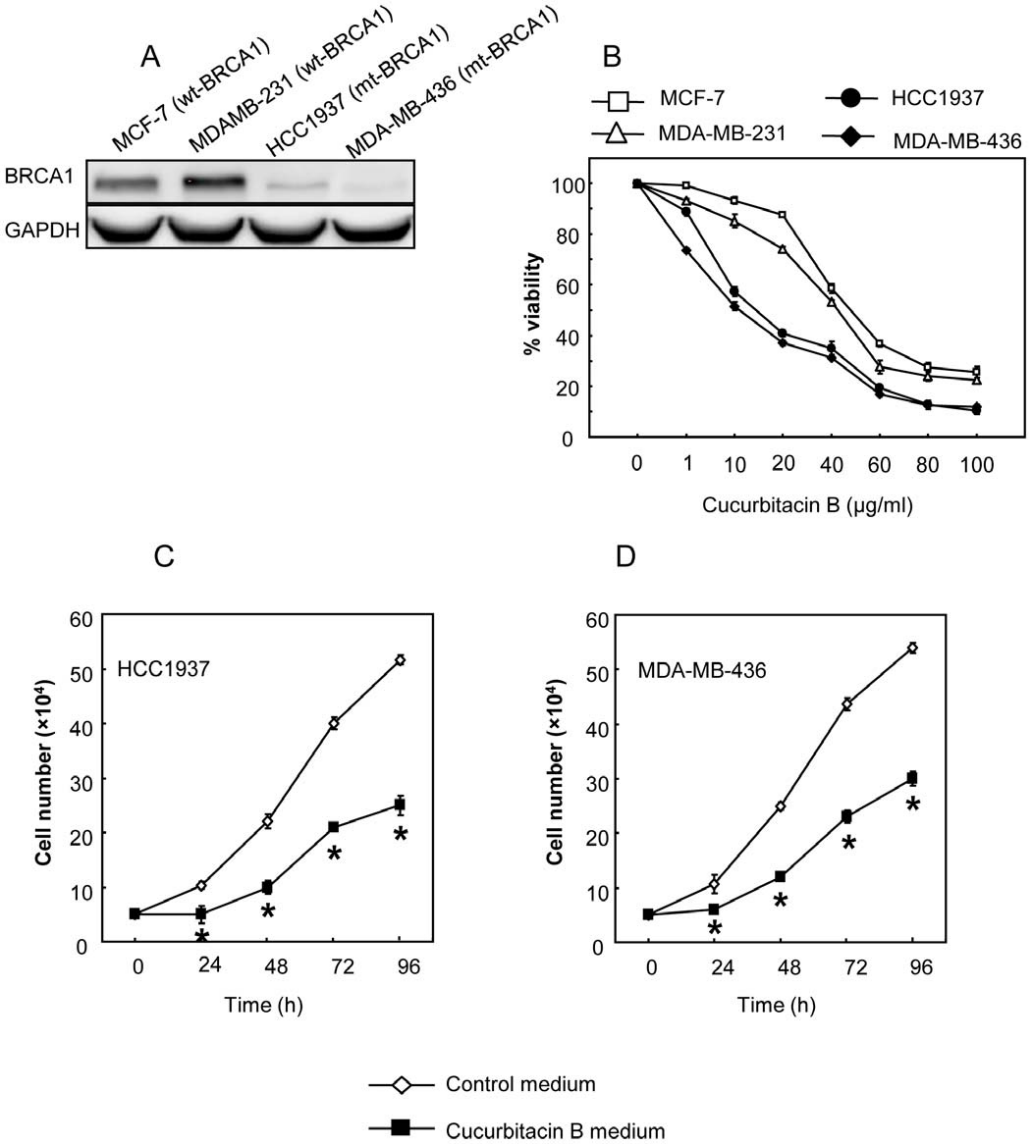
The responses to cucurbitacin B treatment in endogenously expressed wt-BRCA1 breast cancer cells (MCF-7 and MDA-MB-231) and in the two different mutant BRCA1 breast cancer cells (MDA-MB-436 and HCC1937). (A), Western blot analysis for BRCA1 of wild type BRCA1 cells and cells harboring mutant BRCA1. From left to right, MCF-7, MDA-MB-231, HCC1937 and MDA-MB-436, respectively. (B), The cytotoxic effect of cucurbitacin B on endogenously expressed wt-BRCA1 and mutant BRCA1 cells. Each cells were treated with 1, 10, 20, 40, 60, 80 and 100 µg/ml cucurbitacin B for 48 h. (C) and (D), Proliferative rate of the mutant BRCA1 cells after treated with 15 µg/ml cucurbitacin B showed significantly decreased compared with untreated cells, (* *p*<0.01).

**Figure 6:**
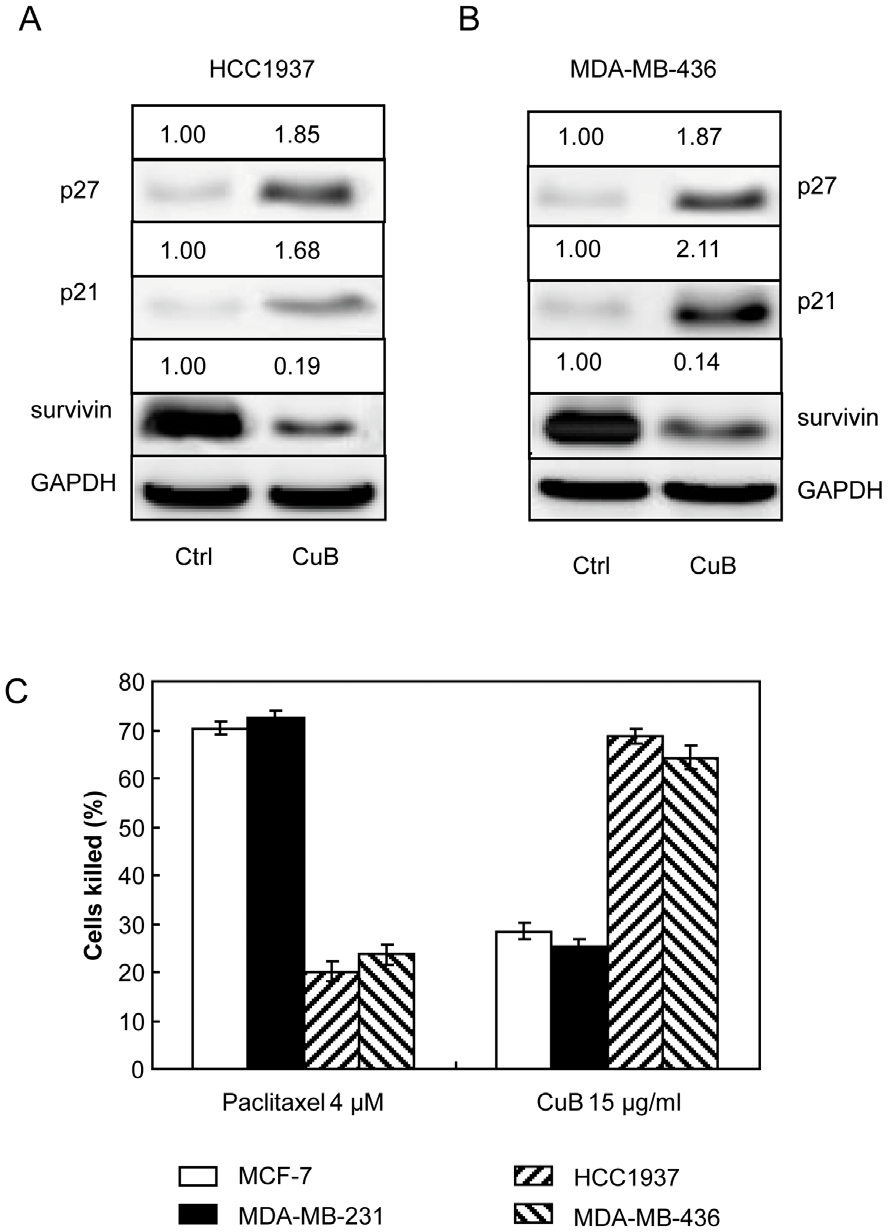
Cucurbitacin B treatment in a mutant BRCA1 breast cancer cells. (A) and (B), Western blot analysis for p21^/WAF1^, p27^kip1^ and survivin in the mutant BRCA1 cells, HCC1937 and MDA-MB-436, after cultured in control medium or in the medium containing 15 µg/ml cucurbitacin B for 48 h. GAPDH was used as loading control. (C), The cytotoxic responses of the wild type and the mutant cells to paclitaxel and cucurbitacin B are shown.

The two BRCA1-defective breast cancer cells (HCC1937 and MDA-MB-436) shown to express low BRCA1 compared to the wild type cells (Fig. 5A). Similar to the BRCA1 knocked-down cells mentioned earlier, cucurbitacin B could suppress the growth of the mutant cells (Fig. 5B). IC_50_ of the BRCA1 mutant cells treated with cucurbitacin B is shown in Table 1. Under cucurbitacin B treatment, both mutant cell types possessed a magnificent lower growth rate (Fig. 5C, 5D) with reduced cell viability in dose dependent manner (Fig. 5B). Significantly increased p27^Kip1^ and p21/^Waf1^ and reduced survivin expressions in the treated mutant cells are shown (Fig. 6A, 6B). By comparison to the wt-BRCA1 breast cancer cells, the mutant cells HCC1937 and MDA-MB-436 expressed higher level of survivin with reduced sensitivity to paclitaxel, indicating as decreased % killed [26]. In contrast, increased sensitivity to cucurbitacin B was clearly observed in BRCA1 deficit mutant cells (Fig. 6C). These results imply that paclitaxel treatment is more effective in the breast cancer cells harboring functional BRCA1 while cucurbitacin B is suitable for the cancer cells with defective BRCA1.

### Mutated BRCA1 gene interferes function of wild type BRCA1 in cellular proliferation

**Figure 7:**
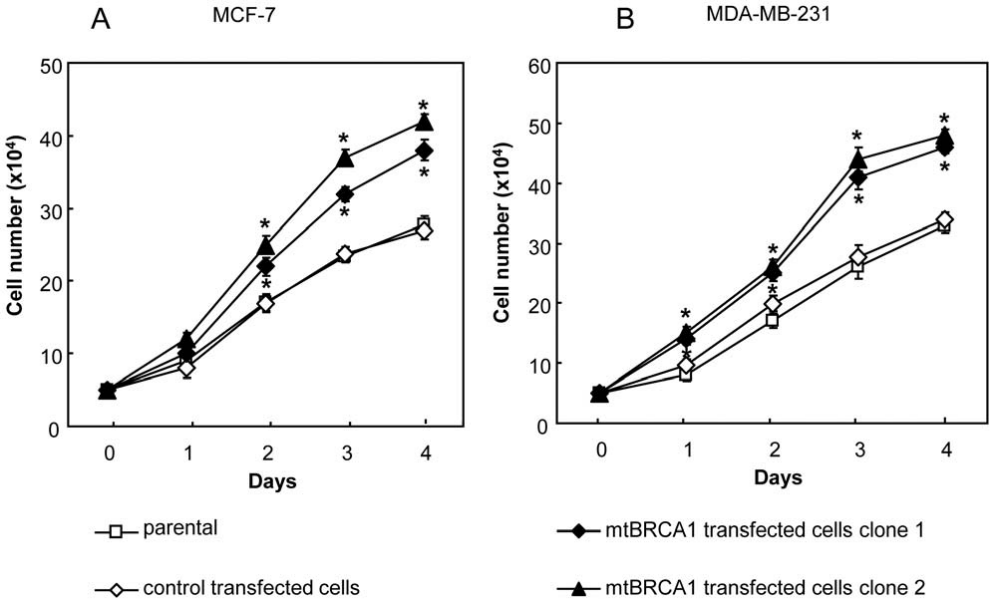
Expression of mutant (Tyr856His) BRCA1 after stably transfected in wild type MCF-7 and MDA-MB-231 cells. (A) and (B), Cell proliferation of MCF-7 and MDA-MB-231 parental, empty vector transfected control and mutant (Tyr856His) BRCA1 transfected cells. Triplicate analysis was performed in each experiment and the values shown represent the mean and standard error of three experiments. Mutant (Tyr856His) BRCA1 transfected cells showed significantly higher proliferative rate compared with the parental cells, (* *p*<0.01).

Stably transfected cells expressing mutated *BRCA1* (Tyr856His) and empty vector transfected (pCEP4) control cells were isolated after selection with hygromycin. The expressions of the transfected mutated *BRCA1* from MCF-7 and MDA-MB-231 cells were confirmed by RT-PCR analysis (not shown). In order to address whether the introduced BRCA1 (Tyr856His) would interfere with tumor suppressor function of wt-BRCA1 in the cells concerning to their cellular proliferation, we then compared the growth rates of breast cancer cells with BRCA1 (Tyr856His) induction with the parental wt-BRCA1 expressing cells. Figure 7A and 7B show the higher proliferative rate of the induced BRCA1 (Tyr856His) mutant cells than the solely wt-BRCA1 parental cells, and the differences were obviously seen as early as 24 hours of culture. The differences were further progressive over the four-day culture.

The BRCA1 (Tyr856His)-transfected mutant cells were also subjected for studying their malignant behaviors (cell migration, invasion and anchorage-independent growth assays). However, the results did not show meaningful difference in these capabilities between the wt-BRCA1 parental cells and the induced BRCA1 (Tyr856His) (data not shown), implying that effect of the introduced BRCA1 point mutation (Tyr856His) gene into the endogenous wt-BRCA1 expressing cells is mild and not enough for influencing the behaviors other than proliferation. By this reason, the induced BRCA1 (Tyr856His) mutant cells thus did not appropriate for studying role of BRCA1 upon paclitaxel and cucurbitacin B treatments. Instead, we selected to study with more suitable BRCA1-defective breast cancer cells (HCC1937 and MDA-MB-436) and shRNA knocked down as reported above.

### Functional BRCA1 abrogates cytotoxic sensitivity to cucurbitacin B of endogenous defective BRCA1 breast cancer cells

**Figure 8:**
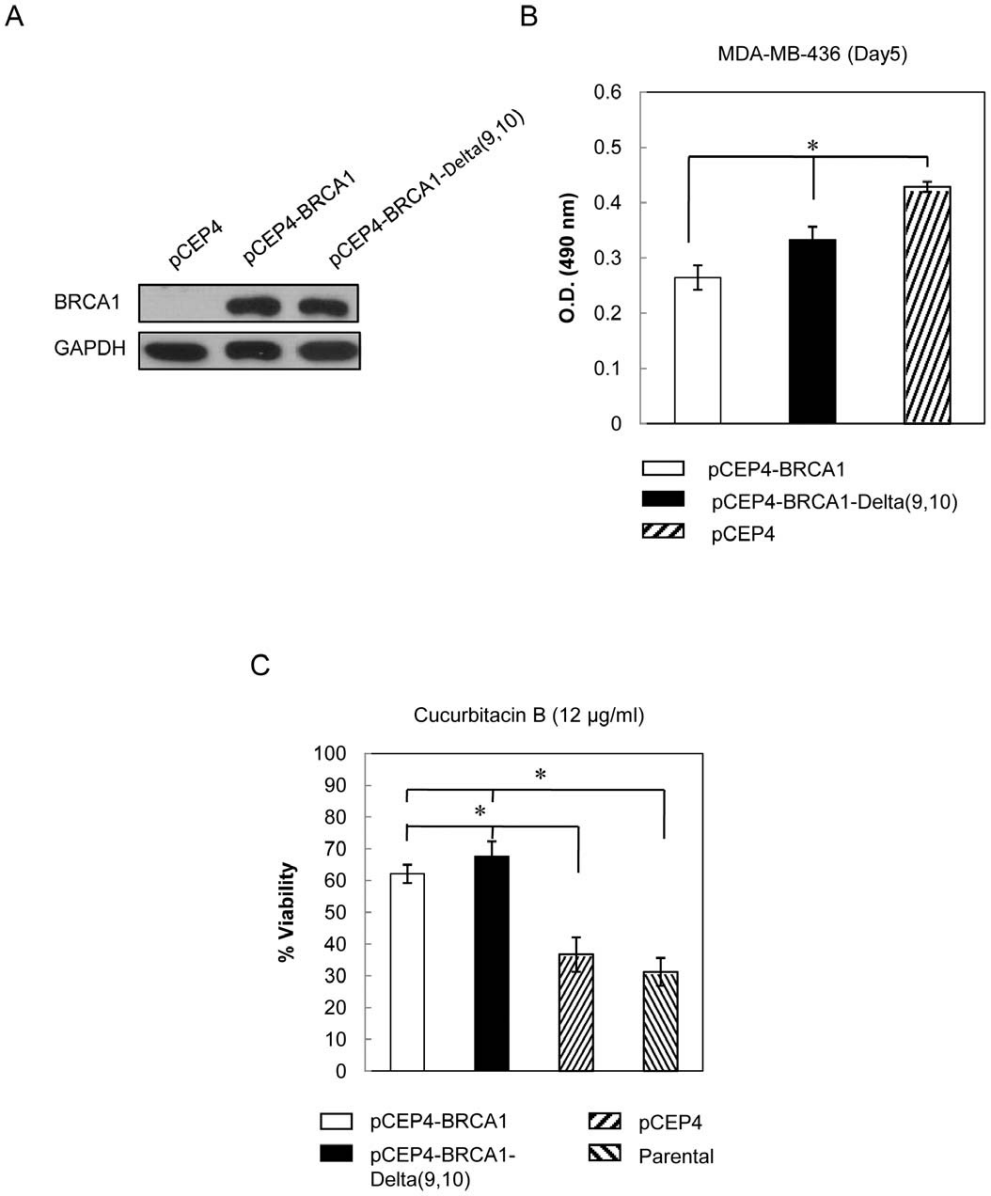
Cucurbitacin B treatment in exogenously induced BRCA1 expressing cells. (A), Western blot analysis for BRCA1 from BRCA1-defective MDA-MB-436 cells which either transfected with vector containing BRCA1 full length (pCEP4-BRCA1) or the splice variant (skip exon 9–10; pCEP4-BRCA1-Delta(9,10)). pCEP4 was used as empty vector control. (B), Cells were grown for 5 days and cell viability was tested by using MTS assay. (C), MDA-MB-436 parental cells, empty vector control cells and cells with transfected BRCA1 were treated with 12 µg/ml cucurbitacin B for 48 h and cell viability was analyzed. BRCA1 expressing cells showed significant higher resistance to cucurbitacin B when compared to the BRCA1 defective parental cells, (* *p*<0.01).

As shown in Figure 6, endogenous BRCA1 defective cancer cells (MDA-MB-436, HCC1937) exhibited higher sensitivity to cucurbitacin B than the wt-BRCA1 expressed cells (MCF-7, MDA-MB-231). We further confirmed the role of BRCA1 on cucurbitacin B sensitivity using exogenous induced BRCA1 expression. Full length BRCA1 vector and the vector containing splice variant BRCA1 Delta(9,10) were stably transfected into BRCA1-defective breast cancer cell, MDA-MB-436. Both the full length BRCA1 and the splice variant encode for functional proteins. Western blots showed the high expression of BRCA1 as compared with empty vector control cells (pCEP4) (Fig. 8A). Cells were then grown for 5 days and cell viability was measured. Both BRCA1 full length and BRCA1 Delta(9,10) could inhibit cell growth when compared to the control cells (Fig. 8B). In order to test cytotoxicity of cucurbitacin B on BRCA1-defective parental and BRCA1-overexpressing cells, each of them were treated with 12 µg/ml cucurbitacin B for 48 hours. The cells having BRCA1 full length and BRCA1 Delta(9,10) were more resistant to cucurbitacin B treatment than the parental and control transfected cells (Fig. 8C).

### Wild type BRCA1 but not mutated BRCA1(3300delA) enhances resistant effect to cucurbitacin B treatment

**Figure 9:**
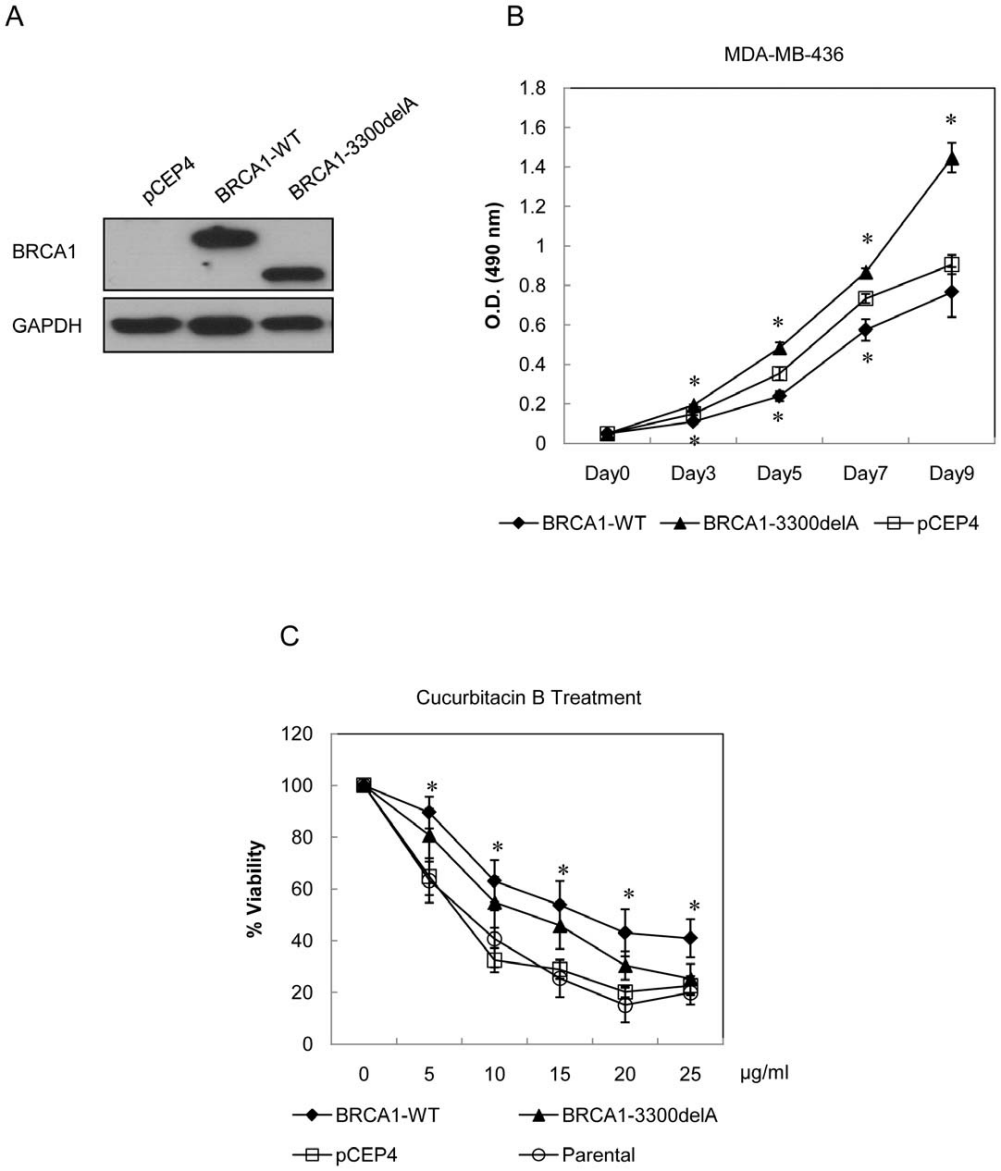
Cucurbitacin B treatment in exoenously induced wt-BRCA1 and mutant BRCA1 expressing cells. (A), Western blot analysis for BRCA1 from BRCA1 defective MDA-MB-436 cells transfected with either wt-BRCA1 vector (pCEP4-BRCA1) or the mutant BRCA1 (3300delA) vector (pCEP4-BRCA1-3300delA). (B), Proliferative rate of wild type and mutant BRCA1 expressing cells. The cells were grown and MTS assay was assessed at indicated times. (C), MDA-MB-436 parental cells, empty vector control cells and cells with wild type or mutant BRCA1 expression were treated with 5, 10, 15, 20 and 25 µg/ml cucurbitacin B for 48 h. Control cells were treated with 0.1% DMSO. The experiments were done in triplicate. The wild type but not mutant BRCA1 expressing breast cancer cells showed significant higher resistance to cucurbitacin B when compared to the parental cells, (* *p*<0.01).

BRCA1 3300delA mutation associates with familial breast cancer in Thai patients [23]. We constructed BRCA1(3300delA) by using BRCA1 full length as a template and both the BRCA1(3300delA) and the full length inserted vectors were stably transfected into BRCA1-defective breast cancer cells MDA-MB-436. BRCA1 expression was detected via Western blot analysis. The BRCA1(3300delA)-transfected cells produced truncated BRCA1 protein of 120 kDa while the full length coded for complete BRCA1 of 220 kDa. The empty vector pCEP4 was used for the transfection control (Fig. 9A). The growth rates of breast cancer cells stably transfected with wt-BRCA1 and the mutated 3300delA were analyzed. As compared with the empty vector control cell, the wt-BRCA1 inhibited cell growth while the BRCA1(3300delA) promoted cellular proliferation (Fig. 9B). Cells were then treated with either control medium or specified concentrations of cucurbitacin B for 48 hours and measured for cell viability. The resistance to cucurbitacin B was observed in the wt-BRCA1. The mutated BRCA1 expressing cells (3300delA transfected) and BRCA1-defective parental MDA-MB-436 cells were equally killed at the concentration of 25 ug/ml (Fig. 9C).

## Discussion

Discovery of local medicinal plants provide an important source of the naturally derived new anticancer drug development including Taxol [36], [37]. Cumulative evidences from previous reports showed that cucurbitacin B has anticancer activity in human cancer cells [38], [39]. We previously reported that cucurbitacin B inhibits growth and telomerase activity in breast cancer cell lines (T47D, SKBR-3, and MCF-7). This agent exerts an obvious inhibitory effect in the estrogen receptor (ER)-negative SKBR-3 cells. Cucurbitacin B also inhibits hTERT and c-Myc expression, implying that it exerts an anticancer effect by inhibiting telomerase via down regulating both the hTERT and c-Myc expression in breast cancer cells [17]. Other studies showed that different cucurbitacin species could also modify biological activities of cancer cells. For instance, cucurbitacin B/E glucosides can induce cell cycle arrest at G_2_/M as well as induce apoptosis in MCF-7 and MDA-MB-231 human breast cancer cells [14]. Cucurbitacin I and Q were shown to specifically inhibit STAT3 phosphorylation which contributes to the proliferation of cancer cells [12].

In this work, we elaborate the effects of cucurbitacin B on breast cancer cells. The anticancer bioactivities of cucurbitacin B on the four breast cancer cell lines were determined. Among the two cell lines with endogenous expression of wild type BRCA1 (MCF-7 and MDA-MB-231), MCF-7 cells are ER positive whereas MDA-MB-231 cells are ER negative. The other two cell lines are endogenous mutant BRCA1 breast cancer cells. MDA-MB-436 possessed 5396+1G>A mutation in the splice donor site of exon 20 and has ER negative whereas HCC1937 has the insertion of a cytosine at position 5382 of *BRCA1*. This mutated type frequently observed in Ashkenazi Jewish. HCC1937 has also negative for ER, PR and Her2/neu [34].

Invasion and metastasis are the major interest in the study on bioactivity of drug in cancer. These two cancer behaviors result in the failure of therapeutic intervention and death. Some report showed that cucurbitacin I can inhibit migration of keloid fibroblasts [40] and also reduces the invasiveness of nasopharyngeal carcinoma cell lines with elevate STAT3 activation [41]. However, the biological effects of cucurbitacin compounds on migration and invasion of breast cancer cells and their possible mechanism have not been completely understood. The reduction of cells invasion and migration could partly due to inhibitory effect of cucurbitacin B on cell viability. The other mechanisms may also involve in the effect of cucurbitacin B on these processes. Recently, Duangmano et al. (2012) [42] reported that cucurbitacin B obviously interferes with the microtubule network, which could be one possible reason for the reduced migration and invasion upon cucurbitacin B treatment. We compared the effects of cucurbitacin B in BRCA1 knocked-down cells with the wild type BRCA1 harboring cells. The results indicated that cucurbitacin B inhibits cellular proliferation, migration, invasion and ability of anchorage-independent growth of the BRCA1 knocked-down breast cancer cells while this compound exerts a minimal effect on the wild type BRCA1 breast cancer cells. Results from BRCA1 mutant cells are similar to that of the BRCA1 knocked-down cells. To support these findings, the exogenous wild type BRCA1 was introduced into the BRCA1-defective breast cancer cells, MDA-MB-436. This extra wt-BRCA1 causes the cells to be cucurbitacin B resistant. Both of the BRCA1 full length and the splice variant BRCA1 Delta(9,10) induced the resistant effects. Some mutations of BRCA1 affected sensitivity to chemotherapeutic drug [43], [44]. For example, the missense mutation D67Y BRCA1 RING domain was more susceptible to cisplatin than wild type BRCA1 RING domain protein [43]. Our study showed BRCA1 (Tyr856His)-transfected mutant cells interfered function of wild type BRCA1 by increased cellular proliferation. However, the BRCA1 (Tyr856His)-transfected mutant cells did not show significant difference in cell migration, invasion and anchorage-independent growth assays. Then, we used the other mutations in order to evaluate cucurbitacin B effects. Cells harboring the BRCA1(3300delA) mutation showed highly proliferated phenomenon when compared with empty vector control. Treatment with cucurbitacin B can inhibit cellular proliferation of these mutant cells and the BRCA1-defective parental cells, suggesting that cucurbitacin B could be an effective anticancer agent properly used for BRCA1-defective breast cancer. Some report has shown that BRCA1 mutant breast cells are generally estrogen receptor negative [45]–[47]. Notably, the ERα expression in BRCA1 mutant cells HCC1937 is recovered when the exogenous wild type BRCA1 was introduced into these cells [47]. Our recent works also demonstrated that ER-negative breast cancer cells are more senstive to cucurbitacin B than the ER-positive breast cancer cells [17], [48]. The explanation of how BRCA1 mutant cells are more sensitive of to cucurbitacin B than the cells harboring wild type BRCA1 probably associates with the ER expression.

From above information, we believe that the normal BRCA1 plays crucial roles in maintaining cellular homeostasis of the normal cells. The presence of tumor suppressor BRCA1 induces expression of ER [47] while the ER can subsequently induce c-Myc expression [49]. The c-Myc upregulates telomerase and the cell proliferation increases [50], [51] to keep balanced with anti-proliferative effect of BRCA1. Loss of BRCA1 could thereby lead to reduced ER and c-Myc expression into lower levels. Expression of c-Myc is also induced by β-catenin/TCF of the Wnt signaling [52]–[55]. Our recent report revealed that c-Myc and cyclin D1 were reduced upon cucurbitacin B treatment in wt-BRCA1 possessed, ER (+) MCF-7 cells. The effect of this agent is more serious in the low BRCA1 expressing, ER (−) SKBR-3 cells [48], [56]. Cucurbitacin B is thought to inhibit the movement of β-catenin and galectin-3 to the nucleus, hence down-regulating their Wnt signaling targets such as c-Myc and cyclin D1. In present work, we clearly show that the breast cancer cells harboring various types of defective BRCA1 are more sensitive to cucurbitacin B than the wt-BRCA1 possessed cells. We suggest that increase sensitivity to cucurbitacin B in BRCA1 defective cells is due to more aggressive reduction of the c-Myc by both reduced ER expression (dues to BRCA1 defect) [49], [57] and effect of cucurbitacin B on β-catenin/TCF of the Wnt signaling, which finally reduced c-Myc and cyclin D1 [17], [48].

Overexpression of survivin is associated with poor prognosis in breast cancer [58], [59]. Previous report has shown that BRCA1 is a negative regulator of survivin [26], and we found herein that survivin expression is upregulated in the BRCA1 knocked-down and mutant cells. We also show that cucurbitacin B could inhibit the expression of survivin and could induce expression of both p21/^Waf1^ and p27^Kip1^ in BRCA1 deficient cells. Anticancer effect by cucurbitacin B had been reported [10], [14]. Thoennissen NH *et al.* [10] showed that cucurbitacin B was associated with inhibition of activated JAK2, STAT3 and STAT5 and increased level of *p21^Waf1^* in human pancreatic cancer cells. While, Tannin-Spitz T *et al.* [14] reported treatment of breast cancer cells with cucurbitacin glucoside dephosphorylated PKB, and inhibited survivin. The simultaneous PKB inhibition and STAT3 inactivation is possibly responsible for the observed induction in p21/^WAF1^ expression. PKB inhibition might also lead to reduction in survivin expression [14]. We also believe that, at least in part, the PKB dephosphorylation is probably associated with p21/^Waf1^ and/or p27^Kip1^ expression which could be associated with reduced survivin level. Our data show that cucurbitacin B suppresses the ability of BRCA1 defective cells to grow and migrate which probably because of the decrease in survivin via PKB inhibition, suggesting that this agent has anti-metastatic potential against the cancer cells. Moreover, we believe that cucurbitacin B interferes with apoptosis and cell cycle control machineries since survivin was inhibited while p21/^Waf1^ and p27^Kip1^ were upregulated in the cancer cells with defective BRCA1. The treatment with cucurbitacin B is more effective in BRCA1 defective breast cancer cells than in the wild type BRCA1 breast cancer cells. Results from this work should be useful for the future selective treatment or personalized medicine of human breast cancer.
